# Combination CBD/THC in the management of chemotherapy-induced peripheral neuropathy: a randomized double blind controlled trial

**DOI:** 10.3389/fonc.2025.1590168

**Published:** 2025-10-23

**Authors:** Marisa Weiss, Muath Giaddui, Stephanie Kjelstrom, Joseph Gary, Sara Jane Ward, Jessica Burrell, Katherine Diguilio, Gabrielle Bidas, Ebuwa Erebor, Sam Meske, Lisa Saeed, Sarah Windawi, Katherine Aliano Ruiz, Arezoo Ghaneie, Julianne Hibbs, John Marks, David Holtz, Zonera Ali, Aarthi Shevade, Jennifer Sabol, Robin Ciocca, Eric Zeger, Paul Gilman, Sharon Larson, Shoichi Shimamoto, Diana Martinez

**Affiliations:** ^1^ Lankenau Institute for Medical Research, Wynnewood, PA, United States; ^2^ Lankenau Medical Center, Main Line Health, Wynnewood, PA, United States; ^3^ Breastcancer.org, Ardmore, PA, United States; ^4^ Center for Population Health Research, Main Line Health, Wynnewood, PA, United States; ^5^ College of Population Health, Thomas Jefferson University, Philadelphia, PA, United States; ^6^ Department of Psychiatry, Columbia University Irving Medical Center, New York, NY, United States; ^7^ Department of Neural Sciences, Center for Substance Abuse Research, Temple University Lewis Katz School of Medicine, Philadelphia, PA, United States; ^8^ Department of Neurology, Baylor University Medical Center, Houston, TX, United States; ^9^ New York State Psychiatric Institute, New York, NY, United States

**Keywords:** chemotherapy-induced peripheral neuropathy, cannabinoids, cannabidiol (CBD), delta-9-tetrahydrocannabinol (THC), sensory impairment, cancer

## Abstract

**Introduction:**

Chemotherapy-induced peripheral neuropathy (CIPN) can greatly impair function, leading to disability or truncated treatment in cancer patients. Previous animal studies show that cannabidiol (CBD) and delta-9- tetrahydrocannabinol (THC) can ameliorate CIPN. This study assessed the effect of combined CBD and THC on CIPN symptoms amongst cancer patients treated with taxane- or platinum-based agents.

**Materials and methods:**

This 12-week randomized, double-blind, placebo-controlled trial included participants with nonmetastatic breast, colorectal, endometrial, or ovarian cancer experiencing grade 2–3 CIPN. The active group received CBD (125.3-135.9 mg) combined with THC (6.0-10.8 mg) in gelcaps. The Quality-of-Life Questionnaire-CIPN twenty-item scale (QLQ-CIPN20) sensory subscale was used as the primary outcome. Additional outcomes assessed pain, sleep, and function. Neurologic exams evaluated touch, pressure, and vibration sense. Following the randomized controlled trial, participants were invited to enroll in a 12-week open-label observational study.

**Results:**

Of 230 participants identified, 124 met eligibility, 54 were enrolled, 46 were randomized, and 43 completed 12 weeks of treatment. This was lower than our goal of 100 randomized participants. The mean age was 60 +/- 9 years, 88% were female, 63% had breast cancer. All participants had completed chemotherapy. The primary analysis showed no differences in outcome measures between active and placebo groups, likely due to sample size. Although an increase in bilirubin (one participant in active group, and one in placebo) and alkaline phosphatase (one participant in active group) was seen, this did not exceed the exit criteria. A secondary analysis showed that the active group experienced greater improvement in the QLQ-CIPN20 measures of sensory impairment relative to placebo (-10.4 (95% -20.5, -0.3), p = 0.044). There was also improvement in light touch and vibration sensation of the feet on neurological exam that approached significance. There was no effect on other measures, including pain, and no between-group differences in side effects. The uncontrolled observational study showed similar results.

**Discussion:**

The primary analysis showed no between-group difference in CIPN symptoms. The secondary analysis indicated that CBD with THC could improve sensory impairment and might increase touch and vibration sense, although these findings require confirmation in a future, more fully powered study. Nonetheless, our results show that combination CBD/THC can be safely delivered to participants with CIPN and suggest that these cannabinoids should be further investigated for this indication.

## Introduction

Chemotherapy-induced peripheral neuropathy (CIPN) is a disabling and dose-limiting complication of cytotoxic agents. The symptoms include distal extremity numbness, tingling, pain, and loss of function. CIPN can impair quality of life and impede patients’ ability to complete curative therapy (1, 2). Research shows that 40-70% of patients treated with taxanes (e.g., paclitaxel, docetaxel) or platinum-based agents (e.g., oxaliplatin, cisplatin, carboplatin) develop CIPN (3, 4). Additionally, the rate of CIPN has remained unchanged for decades, globally, without a decrease in prevalence (4).

Previous studies have investigated a range of therapies for this disorder. These include topical agents, gabapentinoids, antidepressants, physical therapy, acupuncture, and more (5). However, to date, only duloxetine has shown level I evidence of efficacy for CIPN (1, 6). Thus, there is a need to develop additional treatments for this common, refractory disorder.

In mice, cannabidiol (CBD) and delta 9- tetrahydrocannabinol (THC) can ameliorate CIPN caused by paclitaxel and oxaliplatin (7–12). Previous studies show that CBD administration prevented the development of mechanical and cold sensitivity in mice treated with paclitaxel through a potential serotonergic mechanism (7), without psychoactive effects or interference with chemotherapeutic efficacy (8). Follow-up studies showed that CBD was also effective against oxaliplatin-associated mechanical sensitivity and synergized when co-administered with THC (9). These preventative effects of cannabinoids on paclitaxel-associated mechanical sensitivity in mice have also been shown by Kalvala et al. (10, 11) Additionally, Ortiz et al. (12) reported that CBD treatment can reverse paclitaxel-induced mechanical sensitivity.

Previous studies in cancer patients have also investigated cannabinoids for CIPN. In 2014, Lynch et al. (13) compared nabiximols (an oral spray of THC and CBD) to placebo in 18 participants with CIPN and found no significant change in CIPN symptoms (13). In contrast, a retrospective study showed that the use of medicinal cannabis in patients treated with oxaliplatin or 5-fluorouracil-based combinations was associated with a decrease in CIPN severity compared to patients not using cannabis (14). An open label trial administered CBD to two groups of patients receiving chemotherapy: oxaliplatin combined with capecitabine or paclitaxel with carboplatin (15). The results showed decreased cold sensitivity to touch and less throat discomfort in participants who received CBD with capecitabine and oxaliplatin, with no effect on pain for either group (15). Meanwhile, a pilot randomized, placebo-controlled trial of oral cannabinoids (300 mg CBD/15mg THC) showed no improvement (16), while a study of topical CBD cream also showed a lack of an effect on pain (17).

Our goal was to investigate hemp-derived cannabinoids for CIPN using a randomized, double-blind, placebo-controlled design of 12 weeks duration. We enrolled participants with cancer who developed CIPN following taxane- or platin-based chemotherapy. The active medication consisted of gelcaps with cannabinoids that contained CBD combined with THC. Each gelcap contained between CBD (13.9 and 15.1 mg) and THC (between 0.67 and 1.20 mg) and the dose was titrated to 3 gelcaps three times a day. Change in CIPN symptoms was measured with questionnaires while neurologic examination evaluated touch, pressure, and vibration in the extremities. Participants completing the randomized controlled trial were invited to enroll in an open-label, 12-week observational study where the dose was reduced to 1 gelcap twice daily. The outcomes included questionnaires but not neurologic examination.

## Materials and methods

### Randomized controlled trial

This randomized, double-blind, placebo-controlled clinical trial was conducted from 6/1/2020 to 8/8/2022. Approval was obtained from Main Line Hospitals Institutional Review Board and registered with clinicaltrials.gov (NCT04398446). The inclusion criteria were: 1) diagnosed nonmetastatic breast, colorectal, or endometrial cancer or a diagnosis of ovarian cancer (any stage with no evidence of active disease at the time of enrollment); 2) completed treatment with taxane- or platin-based chemotherapy; 3) CIPN of sensory grade 2 or 3 and motor grade < 2 (severity determined using the Common Terminology Criteria for Adverse Events, CTCAE) (18). Exclusion criteria included: 1) current cannabis use (negative urine drug screen required); 2) unstable medical illness and serious psychiatric disorders; 3) history of neuropathy prior to chemotherapy; 4) CIPN of > 2 years; and 5) pregnancy or breastfeeding. Additional details on eligibility criteria are provided in the **Supplementary Material**.

Participants were assigned to active or placebo groups using random blocks of size 2 and 4, with cancer type (breast, colorectal, ovarian/uterine) as the stratification variable. The active medication, delivered in gelcaps, contained CBD (between 13.9 and 15.1 mg per gelcap) and THC (between 0.67 and 1.20 mg per gelcap) while the placebo consisted of hemp-derived product in gelcaps devoid of CBD and THC. The combination of CBD with THC was chosen based on animal data showing synergy when used for CIPN (8). The doses were derived from allometric scaling based on previous animal studies. Because plant-derived cannabinoids were used, there was some variability in the dosages of CBD and THC contained within gelcaps. Details on the dosages selected and the gelcap contents, including the use of third-party testing, are provided in the **Supplementary Material**.

For both groups, the following titration schedule was used: days 1-3, 1 gelcap 3 times daily; days 4-6, 2 gelcaps 3 times daily; day 7 and beyond, 3 gelcaps 3 times daily. Thus, after week 1, participants in the active group took between 125.3 to 135.9 mg CBD daily and between 6.0 to 10.8 mg THC daily. Both active and placebo gelcaps were provided by Ecofibre/Ananda Health and dispensed through an independent pharmacy using codes to maintain blinding. Ecofibre/Ananda Health was not involved in the study design, regulatory approval, research procedures, analysis of data, interpretation of results, or publication of findings.

The following outcomes were measured every 2 weeks for 12 weeks:

1) The primary outcome was the sensory subscale of the European Organization for Research and Treatment of Cancer (EORTC) Quality of Life Questionnaire-CIPN twenty-item scale (QLQ-CIPN20) (19). This questionnaire assesses sensory, motor, and autonomic symptoms: the sensory subscale includes 9 items that measure numbness, tingling, distinguishing temperature, hearing, and pain (19). We also separated the scores for numbness and tingling from pain from the sensory questions so that these items could be investigated independently (questions 31-34, 39, 40 were averaged for numbness/tingling; questions 35 and 36 were averaged for pain). This approach has precedent in CIPN research (20), though it is not formally validated as a primary endpoint.

Additional outcomes included:

2) Neurological examinations were performed using the following assessments (see **Supplementary Material** for additional details):

- The Semmes-Weinstein monofilament examination, which evaluates touch and pressure sensation (21), was performed five times on the palm using five sizes of monofilament, and five times on the big toe using one size of monofilament (see **Supplementary Material** for monofilament sizes). Delivery of the monofilament was alternated in a non-regular pattern with sham delivery. The outcome measure was the number of times the monofilament was accurately detected.
- A 128Hz tuning fork to measure vibratory sensation of the big toe, performed five times (with alternating vibration and no vibration) measured the number of times participants accurately detected vibration or no vibration.
- A 40g semi-sharp sterile tip to measure pinprick pain sensation of the big toe was performed five times. Actual pinprick was alternated (in a non-regular pattern) with sham pinprick. The outcome was the number of times the pinprick was accurately detected by the participant.

3) The Brief Pain Inventory short form (BPI-SF) questionnaire was used to gauge pain (22). The outcomes included the worst and average pain in the past 24 hours (questions 3 and 5) while pain severity was reported as the average of questions 3-6. The pain interference score was obtained using the total score of items 9a-9g.

4) The EORTC QLQ-C30 Quality of Life Survey (QLQ-C30) was used to assess global health status, including physical, psychological and social function (23). The outcomes included a summary score and global health QOL score (23, 24).

5) The PROMIS Sleep Disturbance Scale was used to rate sleep-related impairment (sleep quality, sleep depth, and restoration) (25). The score provides a standardized T-score with a mean of 50 and a standard deviation of 10.

In addition, side effects were assessed at each participant visit and liver function tests were obtained at baseline followed by every 4 weeks for 12 weeks total.

### Statistical analysis (randomized controlled trial)

Baseline patient characteristics, demographics, CIPN grade, cancer type and stage, chemotherapy type, time since completion of chemotherapy and primary outcome results were compared between the two arms at baseline. Continuous variables were summarized using means (standard deviation) if normally distributed, or medians (interquartile range) if non-normally distributed and compared between the two arms using two-sample t-tests (for normal distributions) or Wilcoxon rank-sum tests (non-normal distributions). Categorical variables were summarized using frequencies and percentages and compared between the two arms using Chi-square tests of independence.

The primary data analysis investigated the change from baseline to week 12 and included only participants with complete data (n=20 placebo, n=23 active). The primary outcome was change in the QLQ-CIPN20 sensory subscore and secondary outcomes were change in numbness and tingling (from the QLQ-CIPN20), pain (from the QLQ-CIPN20), other QLQ-CIPN20 sub-scores (motor, autonomic), neurological exams, BPI, QLQ-30, and PROMIS from baseline to week 12. The mean differences from baseline and week 12 were compared between each arm with a two-sample t-test and Cohen’s d for effect size. Multiple imputation was not performed for the primary analysis, as applying it in a small sample could increase the risk of bias.

To address missing data in the primary analysis, we conducted additional secondary analyses using a modified intention-to-treat approach that included all participants who completed at least 8 weeks of study visits. Multivariable linear mixed effects models were built for each scale, with random intercepts to account for repeated measures. With these models, we compared the outcomes between study arms while adjusting for baseline CIPN grade and number of visits. In addition, we further adjusted for baseline scale values for BPI, QLQ-C30, and PROMIS sleep because of differences at baseline. The interaction between arm and time was also added to the model and the predicted probabilities graphed. The interactions were tested for significance with a Wald test. For the linear mixed models, no adjustments were made for missing assessments and all patients who had at least 8 weeks of follow-up were included. Finally, a sensitivity analysis of the CIPN20 sensory and motor scales was conducted for all patients who had completed chemotherapy 18 months or less at baseline.

All statistical analyses were performed in Stata/MP 17.0 (StataCorp LP., Texas, USA). Significance was assessed at the 0.05 level, 95% confidence intervals (CI) were presented, and all tests are two-sided unless noted otherwise. A sample size calculation was performed (see **Supplementary Material** for details).

### Observational study

Participants who completed the RCT were invited to enroll in an optional 12-week open-label observational study. This study was separated by the RCT by a 4-week washout period. Gelcaps from Ecofibre/Ananda Health were sent directly to participants who took one gelcap twice daily containing CBD (between 13.9 and 15.1 mg per gelcap) and THC (between 0.67 and 1.20 mg per gelcap), as above. The same primary outcomes (sans neurological exam) were measured by digital survey at baseline and weeks 4, 8, and 12.

### Statistical analysis (observational study)

Descriptive statistics were performed. Scores were compared between baseline and week 12 of the observational study, as well as between baseline and week 12 of the RCT (for those enrolled in both studies). Because we saw an effect in the RCT for sensory CIPN20 scores, for this scale only we combined the results of the RCT and the observational study. We used two-sample t-tests and Cohen’s D to compare the RCT placebo and RCT active groups for each of these three timescales. We built linear adjusted main effects regression models, using the RCT placebo group as reference, to account for the correlation between participants, time from baseline, baseline CIPN grade, and other CIPN drugs as covariates. Marginal graphs were created by adding time and active vs. placebo group as an interaction in the mixed effects model.

## Results

### Main study: randomized controlled trial

**Figure 1:**
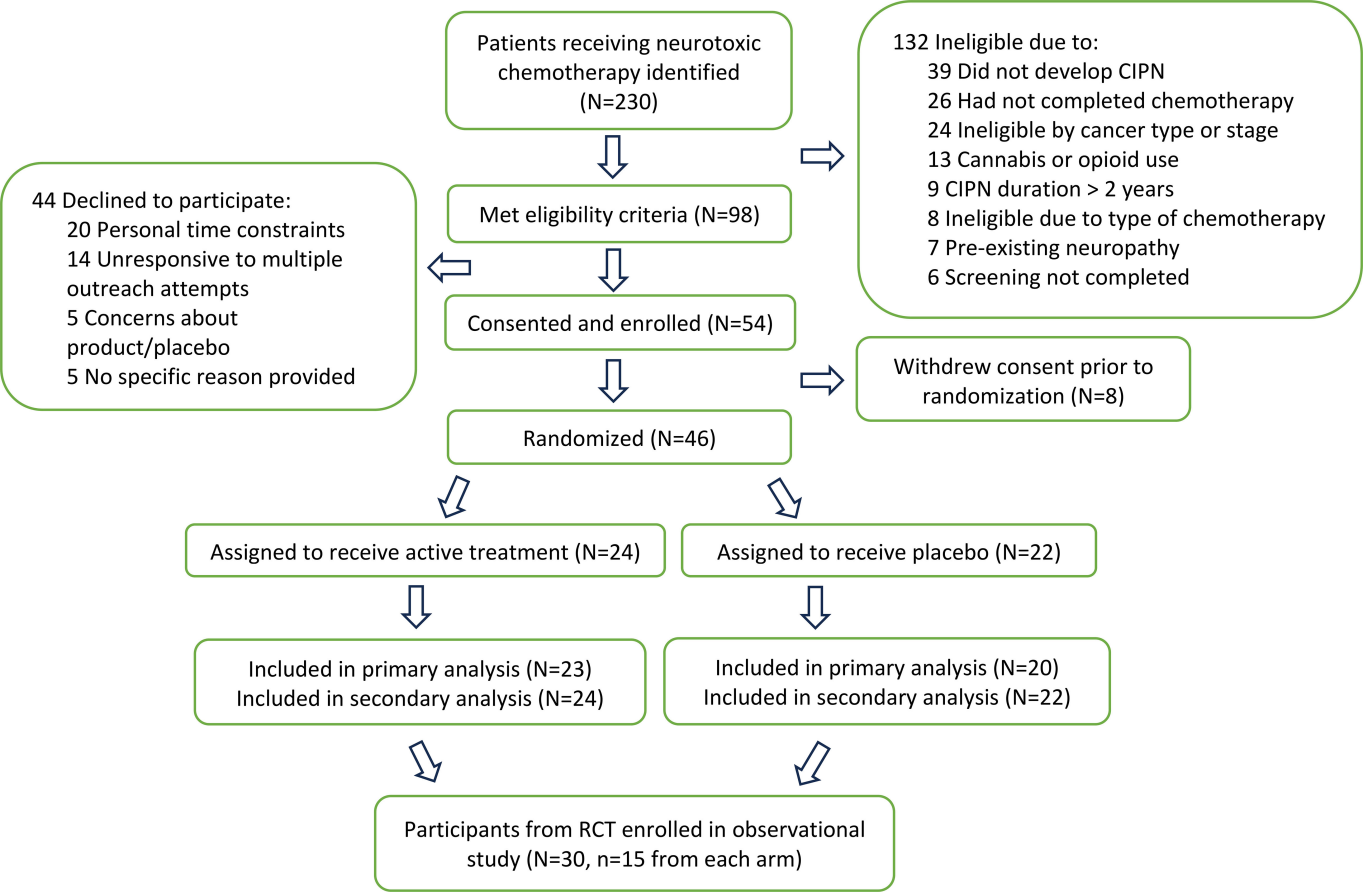
Participant recruitment and enrollment in the RCT and observational study.

Of 230 potential participants, 124 met eligibility criteria and 54 were consented and enrolled. Of these, 46 were randomized and 43 completed 12 weeks (23 assigned to the active group, 20 to placebo) as shown in **Figure 1**. Demographic information and participant characteristics are shown in **Table 1** for those who completed 12 weeks. The median time from chemotherapy (in months) was 3.2 (IQR 1.5-18.9) for the placebo group and 3.6 (IQR 2.7-10.9) for the CBD group. No between-group differences were seen.

**Table 1:** Participant characteristics and demographics by group (n=46).

	Placebo	CBD	Total	p
	n = 20	n = 23	n = 43	
Age (Mean/SD)	61.6 (9.6)	58.6 (8.0)	60 (8.8)	0.272
Sex				0.52
Male	3 (15.0%)	2 (8.7%)	5 (11.6%)	
Female	17 (85.0%)	21 (91.3%)	38 (88.4%)	
Race				0.629
White	14 (70.0%)	16 (69.6%)	30 (69.8%)	
Black	6 (30.0%)	6 (26.1%)	12 (27.9%)	
Other	0 (0%)	1 (4.4%)	1 (2.3%)	
Ethnicity				0.177
Hispanic	0 (0%)	2 (8.7%)	2 (4.7%)	
Not Hispanic	20 (100%)	21 (91.3%)	41 (95.4%)	
Cancer Type				0.513
Breast	13 (65.0%)	14 (60.9%)	27 (62.8%)	
Colon	2 (10.0%)	5 (21.7%)	7 (16.3%)	
Ovarian	4 (20.0%)	3 (13.0%)	7 (16.3%)	
Rectal	1 (5.0%)	0 (0%)	1 (2.3%)	
Uterine	0 (0)	1 (4.4%)	1 (2.3%)	
Chemotherapy				0.694
Carboplatin	2 (10.0%)	1 (4.4%)	3 (7.0%)	
Cisplatin	1 (5.0%)	0 (0%)	1 (2.3%)	
Docetaxel	0 (0%)	2 (8.7%)	2 (4.7%)	
Oxaliplatin	3 (15.0%)	4 (17.4%)	7 (16.3%)	
Paclitaxel	6 (30.0%)	8 (34.8%)	14 (32.6%)	
Docetaxel, Carboplatin	0 (0%)	1 (4.4%)	1 (2.3%)	
Paclitaxel, Carboplatin	6 (30.0%)	6 (26.1%)	12 (27.9%)	
Paclitaxel, Doxorubicin	1 (5.0%)	1 (4.4%)	2 (4.7%)	
Paclitaxel, Doxorubicin, Carboplatin	1 (5.0%)	0 (0%)	1 (2.3%)	
Time from Chemo (Months, Median/IQR)	3.2 (1.5-18.9)	3.6 (2.7-10.9)	3.4 (1.9-10.9)	0.922
CIPN Sensory Grade				0.094
2	20 (100%)	20 (86.9%)	40 (93.0%)	
3	0 (0%)	3 (13.1%)	3 (7.0%)	

CIPN, chemotherapy induced peripheral neuropathy; IQR, Intequartile Range; SD, standard deviation.

The result of the primary analysis is shown in **Table 2** which compared outcomes at baseline, week 12, and change from baseline to week 12. No group differences were observed except the BPI score of pain interference, where the placebo group reported greater improvement compared to the active group (10.9 vs. 0.9, p=0.01). However, there was a difference in the baseline pain interference score between the two groups (26.6 (SD 22.3) vs. 11.8 (SD 15.1), p = 0.011). After adjustment for baseline score, no difference was observed.

**Table 2:** RCT: Comparison of outcomes at baseline, week 12, and change (baseline - week 12).

Mean (SD)	Placebo			Active			p	Cohen's D
	Baseline	Week 12	Change from Baseline*	Baseline	Week 12	Change from Baseline*		
QLQ-CIPN20								
Sensory	42.9 (18.1)	33.3 (22.3)	9.3 (10.3)	40.2 (20.3)	26.4 (22.8)	13.5 (12.5)	0.24	-0.37
Numbness and Tingling	51.5 (16.6)	38.9 (2.6)	15.6 (20.2)	48.4 (21.7)	31.6 (25.0)	16.4 (16.6)	0.88	-0.03
Pain in Hands/Feet	29.5 (31.7)	25 (29.4)	5.6 (24.3)	28.5 (29.7)	21.0 (24.7)	6.5 (17.9)	0.88	-0.1
Motor	32.2 (24)	24.0 (21.0)	6.7 (16.0)	31.3 (24.1)	20.5 (25.0)	10.8 (10.0)	0.31	-0.31
Autonomic	18.2 (19.2)	13.3 (19.2)	2.5 (22.5)	18.1 (27.0)	8.7 (19.4)	9.8 (18.3)	0.25	-0.36
Neurological Exams								
Monofilament								
Palm of Hand Total	19.1 (2.1)	19.9 (1.2)	-0.7 (2.3)	19.2 (3.5)	20.4 (1.8)	-1.3 (3.8)	0.5	0.2
Size 6.65	5 (0)	5 (0)	0	4.9 (0.4)	5 (0)	-0.1 (0.4)	0.36	0.3
Size 5.07	5 (0)	4.9 (0.2)	0.05 (0.2)	4.8 (0.8)	5 (0)	-0.3 (0.9)	0.13	0.5
Size 4.56	4.7 (0.7)	5 (0)	-0.3 (0.7)	4.7 (1.0)	4.9 (0.4)	-0.3 (0.7)	0.86	-0.1
Size 4.31	4 (1.2)	4.7 (0.9)	-0.6 (1.3)	4.5 (1.2)	4.7 (0.8)	-0.3 (1.2)	0.38	-0.3
Size 2.83	0.4 (0.9)	0.3 (0.7)	0.2 (1.2)	0.3 (1.0)	0.8 (1.3)	-0.5 (1.7)	0.17	1
Big Toe 5.07	3.0 (2.1)	3.6 (2.1)	-0.5 (2.2)	3.2 (1.9)	4.5 (1.4)	-1.4 (2.0)	0.15	0.4
Tuning Fork								
Big Toe	3.2 (1.5)	2.8 (2.0)	0.3 (1.6)	3.7 (1.8)	4 (1.4)	-0.3 (1.3)	0.2	0.4
Pinprick								
Big Toe	3.3 (1.3)	3.1 (1.6)	0.2 (1.8)	3.4 (1.4)	3.5 (1.1)	-0.2 (1.5)	0.45	0.2
BPI-SF								
Worst Pain	4.6 (2.7)	3.6 (3.1)	1.1 (2.2)	3.5 (2.9)	3.2 (3.0)	0.2 (2.0)	0.19	0.41
Average Pain	3.5 (2.6)	2.8 (2.1)	0.8 (2.5)	2.3 (2.2)	2.5 (2.5)	-0.2 (2.4)	0.2	0.4
Pain Severity	3.3 (2.4)	2.3 (2.0)	1 (1.8)	2.4 (2.1)	2.3 (2.3)	0.2 (1.9)	0.15	0.45
Interference	26.6 (22.3)	16.0 (18.7)	10.9 (15.2)	11.8 (15.1)	11.3 (16.7)	0.9 (8.3)	0.01	0.8
QLQ-C30								
Summary Score	70.3 (18.7)	78.9 (16.3)	-7.9 (9.4)	79.2 (15.7)	84.9 (14.9)	-5.9 (7.0)	0.46	-0.23
Global Health QOL	57.2 (19.5)	67.9 (20.5)	- 11.3 (18.4)	68.8 (20.9)	73.6 (20.2)	- 4.3 (26.0)	0.33	-0.3
PROMIS Sleep								
Sleep T Score	54.1 (8.3)	51.2 (6.9)	3.3 (7.0)	50.4 (7.9)	49.9 (7.1)	0.98 (8.8)	0.13	0.28

Better clinical outcomes are correlated with higher scores for some scales and lower scores for others. Higher scores indicate better clinical outcomes for the neurological exam, and the QLQ-C30 (summary score and Global Health QoL). Lower scores indicate better clinical outcomes for QLQ-CIPN20, BPI-SF, and PROMIS Sleep. *Change from Baseline = Baseline – Week 12.

SD, standard deviation.

The secondary analysis included 46 participants who completed at least 8 weeks (see **Supplementary Material** for demographic information). The results are shown in **Table 3**. Here, greater improvement was seen in the active group in the sensory score of the QLQ-CIPN20 relative to the placebo group (-10.4 (95% CI -20.5, -0.3), p = 0.044). Additionally, the active group reported greater improvement in numbness and tingling compared to the placebo group (-10.5 (95% CI -20.9, -0.1), p = 0.048) with no difference in pain. No differences were seen in motor or autonomic symptoms. There were no differences in BPI-SF, QLQ-C30, or PROMIS scores. No adjustments were made for multiple comparisons in secondary analyses; as such, these findings should be considered hypothesis-generating and interpreted with caution.

**Table 3:** RCT: Adjusted main effects of primary outcome measures.

Active vs Placebo	Adjusted Main Effects	
	Coeff. (95% CI)	p
QLQ-CIPN20 (a)*		
Sensory	-10.4 (-20.5, -0.3)	0.044
Numbness and Tingling	-10.5 (-20.9, -0.1)	0.048
Pain in Hands/Feet	-7.9 (-21.8, 6.0)	0.268
Motor	-8.5 (-20.1, 3.2)	0.153
Autonomic	-4.1 (-13.9, 5.8)	0.42
Neuro Exams (a)		
Monofilament		
Palm of Hand Total	0.4 (-0.8, 1.5)	0.548
Hand 6.1	0.0 (-0.03, 0.03)	0.999
Hand 5.07	-0.1 (-0.2, 0.1)	0.374
Hand 4.56	-0.01 (-0.2, 0.2)	0.941
Hand 4.31	0.3 (-0.1, 0.8)	0.133
Hand 2.86	0.2 (-0.1, 0.5)	0.174
Big Toe 5.07	0.9 (-0.003, 1.7)	0.051
Tuning Fork	0.8 (-0.01, 1.5)	0.053
Pinprick	0.4 (-0.2, 1.0)	0.17
BPI (b)*		
Worst Pain	-0.1 (-0.9, 0.7)	0.748
Average Pain	-0.04 (-0.8, 0.7)	0.916
Pain Severity	-0.09 (-0.7, 0.6)	0.781
Pain Interference	1.7 (-2.9, 6.3)	0.478
QLQ-C30 QoL (b)		
Summary Score	-0.9 (-4.3, 2.4)	0.587
Global Health QOL	2.1 (-2.4, 6.7)	0.362
PROMIS Sleep (b)		
Sleep T Score	-2.6 (-6.0, 0.9)	0.14

Coefficients are for active vs. placebo.

a. Adjusted for time in study and baseline CIPN Grade.

b. Adjusted for time in study, baseline CIPN grade, and baseline score.

* Negative Coefficients indicate better clinical improvement for active group relative to placebo.

The results of the neurologic exam are also shown in **Table 3**. Monofilament testing of the big toe (0.9 (95% CI -0.003, 1.7), p=0.051) and tuning fork testing (0.8 (95% CI -0.01, 1.5), p=0.053) approached significance for improvement in the active group relative to placebo. No differences were found in the pinprick exam of the big toe or monofilament testing of the hand.

The side effects reported by participants are shown in **Table 4**. No between-group differences were seen. There were no serious adverse events, and no participant was removed from the study due to an adverse event. Please see **Supplementary Material** for side effects categorized by severity using CTCAE criteria (**Supplementary Tables S2**, **S3**). As shown in these tables, fatigue, gastrointestinal distress, and sleep disturbance were the most common side effects. The majority of side effects were rated as grade 2 (moderate symptoms).

**Table 4:** RCT: Side Effects reported by participants by group (active and placebo).

Side Effect	Active	Placebo	p
	n = 24	n = 22	
Fatigue	9 (37.5%)	10 (45.5%)	0.765
GI Distress/Indigestion	4 (16.7%)	2 (9.1%)	0.667
Nausea	3 (12.5%)	1 (4.5%)	0.609
Skin Irritation	0	1 (4.5%)	0.478
“Doesn’t like how it feels”	1 (4.2%)	1 (4.5%)	0.999
Lightheadedness	3 (12.5%)	1 (4.5%)	0.609
Sleep disturbance	2 (8.3%)	3 (13.6%)	0.659
Increased Sweating	1 (4.2%)	0	0.999
Positional vertigo	1 (4.2%)	1 (4.5%)	0.999
Increased appetite	2 (8.3%)	1 (4.5%)	0.999
Feels “foggy”	1 (4.2%)	0	0.999
Hot flashes	2 (8.3%)	0	0.49
Dry Eyes/Mouth	0	1 (4.5%)	0.478
Migraine/Headaches	0	1 (4.5%)	0.478
High Bilirubin	1 (4.2%)	0	0.999
High Alkaline Phosphatase	1 (4.2%)	1 (4.5%)	0.999

Of note, two participants in the active group experienced an increase in lab values (one bilirubin and one alkaline phosphatase) while one in the placebo group had an increase in bilirubin. No participant had an increase in lab values that exceeded two times normal and met criteria for early discontinuation.

The sensitivity analysis including only patients whose time from chemotherapy at baseline was 18 months or less included 17 placebo and 21 CBD. The results were similar to the primary analysis (see **Supplementary Tables S4**, **S5** in the **Supplementary Material**).

### Observational study (following the RCT)

A total of 30 participants were enrolled in the observational study. Of these, 15 had previously been assigned to the active group and 15 were in the placebo group. The time between the RCT and observational study was 28–92 days, which could have impacted the outcomes. Participant demographic information and characteristics are shown in **Table 5**, with the participants separated by the group assigned to in the RCT. No differences were seen between the two groups.

**Table 5:** Observational study: Participant characteristics and demographics by group in the observational study (separated by the group assigned to in the RCT).

	RCT Placebo	RCT Active	Total	p
	n = 15	n = 15	n = 30	
Age (Mean/SD)	63.3 (8.9)	58.1 (9.1)	60.7 (9.2)	0.124
Sex				0.999
Male	2 (13.3%)	2 (13.3%)	4 (13.3%)	
Female	13 (86.7%)	13 (86.7%)	26 (86.7%)	
Race				0.69
White	11 (73.3%)	10 (66.7%)	21 (70.0%)	
Black	4 (26.7%)	5 (33.3%)	9 (30.0%)	
Other	0 (0%)	0 (0%)	0 (0%)	
Ethnicity				0.309
Hispanic	0 (0%)	1 (6.7%)	1 (3.3%)	
Not Hispanic	15 (100%)	14 (93.3%)	29 (96.7%)	
Cancer Type				0.7
Breast	10 (66.7%)	8 (53.3%)	18 (60.0%)	
Colon	3 (20.0%)	3 (20.0%)	6 (20.0%)	
Ovarian	2 (13.3%)	3 (20.0%)	5 (16.7%)	
Rectal	0 (0%)	0 (0%)	0 (0%)	
Uterine	0 (0%)	1 (6.7%)	1 (3.3%)	
Chemotherapy Type				0.367
Carboplatin	2 (13.3%)	1 (6.7%)	3 (10.0%)	
Cisplatin	1 (6.7%)	0 (0%)	1 (3.3%)	
Docetaxel	0 (0%)	1 (6.7%)	1 (3.3%)	
Oxaliplatin	3 (20.0%)	3 (20.0%)	6 (20.0%)	
Paclitaxel	5 (33.3%)	2 (13.3%)	7 (23.3%)	
Docetaxel, Carboplatin	0 (0%)	1 (6.7%)	1 (3.3%)	
Paclitaxel, Carboplatin	3 (20.0%)	6 (40.0%)	9 (30.0%)	
Paclitaxel, Doxorubicin	0 (0%)	0 (0%)	0 (0%)	
Paclitaxel, Doxorubicin, Carboplatin	1 (6.7%)	0 (0%)	1 (3.3%)	
CIPN Sensory Grade				0.143
2	15 (100.0%)	13 (86.7%)	28 (93.3%)	
3	0 (0%)	2 (13.3%)	2 (6.7%)	

RCT placebo, participant was assigned to placebo group in the RCT; RCT Active, participant assigned to the active group in the RCT; SD, standard deviation.

The analysis of the observational study compared participants by their previously assigned group in the RCT (referred to as RCT placebo or RCT active). The results (**Table 6**) showed that the RCT active group reported an improvement in the QLQ-CIPN20 sensory score, and numbness/tingling compared to the RCT placebo group, with no change in pain. Within each group, there was no significant change for other outcome measures in the observational study only. No participants required removal from the study due to adverse events.

**Table 6:** Observational study: comparison of outcomes at baseline, week 12, and change (baseline - week 12).

Mean (SD)	RCT Placebo			RCT Active			p	Cohen's D
	Baseline (n=15)	Week 12 (n=15)	Change from Baseline *	Baseline (n=15)	Week 12 (n=15)	Change from Baseline *		
QLQ-CIPN20								
Sensory	34.6 (23.4)	37.8 (20.2)	-3.2 (12.5)	23.5 (14.2)	18.3 (14.8)	5.2 (10.2)	0.005	-0.7
Numbness and Tingling	39.3 (22.6)	42.6 (21.1)	-3.3 (14.1)	28.9 (15.6)	20.7 (17.2)	8.1 (9.6)	0.014	-0.9
Pain in Hands/Feet	26.7 (32.0)	32.2 (26.3)	-5.5 (322.4)	16.7 (24.4)	16.7 (17.8)	0 (17.8)	0.459	-0.3
Motor	28.9 (23.9)	27.9 (23.9)	0.95 (11.8)	16.2 (18.5)	13.3 (17.3)	2.9 (8.2)	0.612	-0.2
Autonomic	17.8 (23.1)	15.6 (20.4)	2.2 (16.5)	1.1 (4.3)	4.4 (7.6)	-3.3 (6.9)	0.239	0.4
BPI-SF								
Worst Pain	4.6 (3.5)	3.9 (3.6)	0.6 (3.6)	3.9 (3.1)	3.4 (2.7)	0.9 (2.2)	0.763	-0.1
Average Pain	3.6 (2.5)	2.8 (2.7)	0.7 (1.4)	3.3 (3.0)	2.6 (1.8)	1 (2.2)	0.685	-0.2
Pain Severity	3.3 (2.5)	2.7 (2.5)	0.5 (1.8)	2.9 (2.5)	2.4 (1.7)	0.7 (1.7)	0.742	-0.1
Pain Interference	19.9 (19.4)	19.1 (18.8)	0.7 (5.8)	11.9 (16.4)	10.1 (14.4)	1.8 (5.1)	0.598	-0.2
QLQ-C30 QoL								
Summary Score	80.2 (14.8)	77.7 (15.1)	2.4 (6.8)	85.8 (11.0)	87.9 (10.1)	-2.1 (6.3)	0.07	0.7
Global Health QOL	64.4 (20.0)	60.6 (21.4)	3.9 (17.5)	73.3 (15.8)	77.2 (17.1)	-3.9 (12.9)	0.177	0.5
PROMIS Sleep								
Sleep T Score	56.6 (10.8)	56.9 (13.2)	-0.9 (6.1)	54.7 (10.4)	50.2 (10.7)	3.8 (11.8)	0.426	-0.3

Better clinical outcomes are correlated with higher scores for some scales and lower scores for others. Higher scores indicate better clinical outcomes for the neurological exam, and the QLQ-C30 (summary score and Global Health QoL). Lower scores indicate better clinical outcomes for QLQ-CIPN20, BPI-SF, and PROMIS Sleep. *Change from Baseline = Baseline – Week 12.

SD, standard deviation.

## Discussion

In this randomized, placebo-controlled clinical trial of plant-based CBD with THC for CIPN induced by platinum-based or taxane chemotherapy we saw no difference between the active and placebo groups in our primary analysis. This was likely due to the number of participants which did not reach our goal of 100, as indicated by an *a priori* sample size calculation. This may have limited statistical power and increased the risk of Type II error. As such, additional studies with larger samples are needed to validate these findings.

Our secondary, exploratory analysis included all randomized participants (adjusted for time in the study and baseline CIPN grade). It showed that the combination of CBD/THC could improve sensory impairment in CIPN. We saw a reduction in the sensory score of the QLQ-CIPN20 and a decrease in self-reported numbness and tingling in the active condition compared to the placebo. This finding was accompanied by a trend in the results of the neurologic exam, which suggested that the active treatment might increase touch and vibration sense in the feet compared to placebo. However, because of the sample size and the potential for type II errors, however, these findings need to be considered preliminary and in of validation in larger studies.

If validated in a larger study, the finding of improved sensory function has the potential to help patients with CIPN. Numbness and tingling are among the most common symptoms of sensory impairment in cancer patients (20, 26, 27) and lead to significant disability and fall-related injuries (28). However, there remains a lack of treatment options: only duloxetine shows clear evidence of efficacy for CIPN (1, 6), but has a modest impact on numbness and tingling (29).

On neurologic exam, we saw a trend towards improved protective sensation and vibration sense of the feet in the active group over the placebo group. Protective sensation is crucial for normal function and vibration sense is closely tied to the ability to sense position and maintain balance (30). Impaired postural control and loss of balance in CIPN is associated with functional impairment and a risk of physical harm (31).

We saw no serious adverse events and no difference in side effects between the active and placebo groups, indicating that CBD (125.1 to 135.9 mg daily) combined with THC (between 6.0 to 11.5 mg daily) was well tolerated. We did see a small increase in bilirubin and alkaline phosphatase although this occurred in both groups. However, this increase did not surpass the exit criteria of twice normal values for these laboratory measures.

In the open-label observational study, we compared participants who had previously been assigned to the active group in the RCT to those who received placebo in the RCT. These results showed that participants in the active group experienced greater improvement in the QLQ-CIPN20 sensory score and numbness/tingling compared to the placebo group. We also saw no adverse events leading to removal from the study for safety purposes. However, the observational portion of the study included no control group or neurological exams which limits the interpretation of these results.

Our findings add to the literature on cannabinoids for CIPN. An early pilot study investigated nabiximols for CIPN and did not find a significant effect on pain or sensory function, but showed that the medication was well tolerated by cancer patients (13). A more recent placebo-controlled pilot study (n=12) of oral cannabinoids (300 mg CBD/15 mg THC) showed improved CIPN in the placebo group (16). However, a retrospective analysis of patients (700+) treated with oxaliplatin and 5-fluorouracil-based combinations showed that cannabis use was associated with lower CIPN severity and a decrease in the development of neuropathy caused by oxaliplatin (14). A randomized, placebo-controlled trial of topical CBD cream for CIPN showed no effect on pain (17), but a case series reported that topical creams with THC and/or CBD might improve painful CIPN (32).

Recently, Nielsen et al. prospectively investigated the effect of CBD in participants (n=54) scheduled to receive carboplatin and paclitaxel or capecitabine and oxaliplatin (15). The active group received CBD (300 mg daily) administered for 8 days, starting the day before chemotherapy. A non-randomized control group was included who did not receive placebo. The results showed less cold sensitivity to touch, throat discomfort, and discomfort swallowing cold liquids in participants who received CBD (along with capecitabine with oxaliplatin) with no effect on pain or numbness/tingling compared to the control group.

Taken together, the results of these studies, including ours, indicate that CBD and THC are tolerated well in this patient population and could have a clinical use in addressing CIPN. However, the findings are mixed, including the effect on pain. Although previous placebo-controlled studies have shown that THC can reduce painful neuropathy caused by diabetes or HIV (33–36), studies using cannabinoids for CIPN are more mixed. This might result from the CIPN studies using lower doses of THC, which has been shown to reduce pain caused by a range of medical disorders (37). Nonetheless, given that CIPN generally includes numbness and tingling more commonly than pain (20, 26, 27) these studies suggest that cannabinoids may have a future therapeutic role.

The mechanism behind the ability of cannabinoids to improve the sensory symptoms of CIPN is not fully understood but likely includes a range of cellular processes. CBD has a low affinity for cannabinoid receptors, but regulates the endocannabinoid system as an allosteric modulator, and has up to 56 neurological molecular targets (38, 39). We and others have investigated three potential mechanisms of action using *in vivo* and *in vitro* preclinical assays: 5-HT1A receptor antagonism, GPR55 (G protein-coupled receptor 55) receptor antagonism, and the Na^+^-Ca^2+^ exchanger in mitochondria (mNCX).

*In vivo*, antagonism of the 5-HT1A receptor attenuates the neuroprotective effects of CBD in preclinical models of neuropathic pain (40), including CIPN (8). However, direct clinical studies testing 5-HT1A activation specifically for relief of sensory symptoms associated with neuropathies humans have not been performed. An analogue of CBD that is selective for GPR55 receptor antagonism, KLS-13019, effectively attenuates pain and inflammation associated with CIPN *in vivo* as well as *in vitro* (41, 42). We and others have demonstrated that CBD can engage mNCX to regulate intracellular calcium levels (43, 44), including in response to paclitaxel (39). While there is emerging evidence that the mNCX plays a role in pain and sensory function, direct human data is needed.

CBD also interacts with a wide range of other receptors, cellular signaling cascades, and proinflammatory cytokines, and has been shown to reduce oxidative stress and to inhibit proinflammatory pathways (38, 39, 45, 46). While these mechanisms contribute to neuronal damage in CIPN, the exact etiology underlying the sensory disturbances of parasthesias and dysesthesias are not known. As mentioned, Δ9-THC prevents symptoms in CIPN in rodent models and potentiates the effects of CBD; these effects are likely attributed to direct actions on canonical CB1 and CB2 receptors (47). Unfortunately, while biomarkers are being developed to predict and monitor CIPN, there is less evidence for their use in measuring response to specific CIPN treatments.

Overall, this study suggests that combination CBD/THC could help with the sensory impairment seen in CIPN. Since the disorder is prevalent and incurs significant hardship, even a modest sensory improvement could enhance patients’ quality of life, given the lack of alternatives. Further, this study included participants who had completed chemotherapy, suggesting that improvement may occur following the onset of CIPN, which might help some patients. Future studies should include dose-ranging trials, biomarker endpoints, and male-inclusive cohorts to better define therapeutic windows.

## Limitations

A significant limitation of this study was the small sample size. However, we had expected an attrition rate of 30% when the actual rate was 15%, which may reflect the medication being well tolerated. In addition to sample size, other factors could have influenced the results such as suboptimal dosing or delayed timing of the intervention post-chemotherapy. This study included 88% women (12% men), which likely results from the types of cancer diagnoses (63% breast and 16% ovarian). This prevented an assessment of sex-related differences, including metabolism differences (48), and limits the generalizability to males. Baseline time from treatment did not differ between the groups, however, since this study occurred after chemotherapy, the treatment effect may have been attenuated. Future studies may include participants CIPN limited to a shorter duration.

The gelcaps contained cannabis product, which meant that there was variation in the dosages. Although this would be expected when using any plant-derived cannabis product, it could have an impact on reproducibility. Participants were asked to keep a logbook to record compliance with medication, which was close to 100%. However, pill counts were not performed which would have provided a more direct measure.

## Data Availability

The original contributions presented in the study are included in the article/**Supplementary Material**. Further inquiries can be directed to the corresponding author.
