# Supplementary material for: Combination CBD/THC in the management of chemotherapy-induced peripheral neuropathy: a randomized double blind controlled trial
Source: Front Oncol. 2025 Oct 23;15:1590168. doi: 10.3389/fonc.2025.1590168 (PMC12588866; doi:10.3389/fonc.2025.1590168)
Supplement: Supplementary file 1 [file DataSheet1.pdf]

SUPPLEMENTARY MATERIAL

<u>Inclusion and exclusion criteria</u>

- ***Inclusion***
    - Non-metastatic breast cancer patients who developed CIPN (CTCAE sensory grade 2 or 3; motor grade < 2) after receiving taxane-based chemotherapy in pre-operative or post-operative setting;
    - Non-metastatic colorectal cancer patients with high risk stage II and stage III disease who developed CIPN (CTCAE sensory grade 2 or 3; motor grade < 2) after receiving oxaliplatin in the adjuvant setting;
    - Ovarian cancer patients who developed CIPN (CTCAE sensory grade 2 or 3; motor grade < 2) after receiving taxane-containing chemotherapy in the neoadjuvant or adjuvant setting;
    - Non-metastatic uterine cancer patients who developed CIPN (CTCAE sensory grade 2 or 3; motor grade < 2) after receiving taxane-containing chemotherapy in the neoadjuvant or adjuvant setting (with course completed);
    - Non-metastatic pancreatic cancer patients who developed CIPN (CTCAE sensory grade 2 or 3; motor grade < 2) after receiving taxane-containing chemotherapy in the neoadjuvant or adjuvant setting;
      Note: the duration of CIPN was limited to two years to avoid a potential issue with chronic symptoms being less responsive.
- ***Exclusion***
    - Family history of genetic/familial neuropathy;
    - Use of recreational or medicinal marijuana products or use of illegal drugs (per urine drug screen);
    - Use of opioids;
    - Known underlying liver disease (cirrhosis with Child-Pugh score of B or C) or baseline elevation in liver function tests (ALT, AST, total bilirubin) of ≥1.5 x upper limit of normal values;
    - Patients taking these medications were excluded: clarithromycin, itraconazole, erythromycin, fluconazole, clopidogrel, rifampin, sulfamethoxazole, any opioids, warfarin, antiepileptic medications (including carbamazepine, phenytoin, valproic acid, but excepting of clonazepam or diazepam);
    - Underlying history of epilepsy/ recurrent seizure disorder or unexplained seizure within past 6 months;
    - Patients with uncontrolled cardiovascular disease defined by myocardial infarction, stroke, or transient ischemic attack, or need for coronary stent placement within past six months;
    - Patients with uncontrolled psychiatric illness (per DSM-V criteria) or who are at increased risk for suicidality based on baseline Columbia-Suicide Severity Rating Scale;
    - Women who are pregnant, breastfeeding, or not practicing an effective form of birth control.

Sample Size Calculation*:* The calculation was powered to detect longitudinal changes in CIPN, as measured by CIPN20. An effect size of 0.5 standard deviations can be considered a clinically meaningful change in most health-related quality of life studies (Norman et al., 2003). Using a two-sided paired t-test with an alpha level of 0.025, an attrition rate of 30%, and a within-subject correlation of r=0.10 between repeated measurements, random assignment of 50 patients per arm was needed to have 80% power to detect an effect size of at least 0.5 standard deviations on any of the CIPN20 sub-scales. Calculations are based on a study of breast cancer patients by Simon et al., (2017). We assumed baseline scores for ovarian, colorectal, and uterine cancer patients would be similar.

Dosage Selection: The study doses were derived from several bodies of evidence. Firstly, we and others have shown that higher ratios of CBD to THC achieve synergistic results while those more balanced to a 1:1 ratio of CBD to THC lead to additivity or subadditivity as well as the presence of common THC adverse effects (King et al 2017; Br J Pharmacol 2017;174(17):2832–284; Boggs et al, Neuropsychopharmacology 2018 Jan;43(1):142-154; Reisdorph et al, J Cannabis Res 2024 2024 Mar 2;6(1):10). In addition, CBD is effective in CIPN rodent models over a wide range of doses, from 2.5 – 100 mg/kg depending on the study.

When considering allometric scaling of effective CBD doses from rodent studies using a 12.3× conversion factor for body surface area, a 2.5 mg/kg dose in the mouse equals approximately 14 mgs CBD in humans, and a 100 mg/kg dose equals 560 mg CBD. The tested human dose (125–135 mg CBD) represents a conservative adjustment to this model, prioritizing safety and tolerability. Also, THC doses between 6–10 mg align with human studies showing minimal psychoactivity at ≤10 mg when combined with CBD (Englund et al. Neuropsychopharmacology. 2023 May;48(6):869-876).

Gelcap contents: Because the CBD and THC were derived from plant material, slight variation in the content of the gelcaps was present. Certificates of analyses (COAs) were obtained on both active and placebo gelcaps using third-party testing. The COA included measurements of pesticides, microbials, mycotoxin, heavy metals, and solvents, all of which passed the quality control testing for safety.

The COA also analyzed the gelcaps for the presence of other cannabinoids, as follows:

- The active gelcaps contained:
  - Cannabichromene: 0.68 to 1.6 mg per gelcap
  - Cannabidiol: 13.9 to 15.1 mg per gelcap
  - Cannabidivarin: 0.0 to 0.3 mg per gelcap
  - Cannabigerol: 0.1 to 0.22 mg per gelcap
  - Gamma-terpinene: 0.0 to 0.12 mg per gelcap
  - Linalool: 0.0 to 0.075 mg per gelcap
  - Delta-8-THC: 0.0 mg per gelcap
  - Delta-9-THC: 0.67 to 1.2 mg per gelcap
- The placebo gelcaps contained:
  - Cannabidiol: 0.0 mg per gelcap

Cis-oscimene: 0.0 to 0.15 mg/g
Delta-3-Carene: 0.0 to 0.24 mg/g
Delta-8-THC: 0.0 mg per gelcap
Delta-9-THC: 0.0 mg per gelcap

<u>Neurologic Exam</u>: Additional details of the exams included here:
<u>The Semmes-Weinstein monofilament examination:</u> For the hand, this was performed with 5 different monofilaments (sizes 2.83, 4.31, 4.56, 5.07, and 6.65). The filaments were applied to the palm of the hand that was most affected, or the dominant hand if there was no laterality in sensory symptoms. To reduce bias, delivery of the monofilament was interspersed with sham delivery (with participant blinded) in a non-regular pattern. Each monofilament was delivered 5 times and sham was delivered 5 times. Participants were asked whether the filament was perceived (yes/no) and the outcome measure was the number of times the participant accurately reported the monofilament delivery (actual or sham).

For the foot, one filament size was used (5.07) and applied to the big toe of the most affected side (if not different, the dominant side was used). Delivery of the monofilament was interspersed with sham delivery (with participant blinded to the condition) in a non-regular pattern (each condition delivered 5 times). Participants were asked whether the filament was perceived (yes/no) and the outcome measure was the number of times the participant accurately reported the delivery of the monofilament (actual or sham).

<u>Tuning fork for vibration sense:</u> A 128Hz tuning fork was applied to the dorsal surface of the last knuckle (interpharyngeal joint) big toe. The examiner alternated between producing a vibration (by tap tuning fork against the palm of his/her hand) and no vibration. The tuning fork was applied 5 times. Participants were blinded to active versus sham conditions. Participants were asked whether the filament was perceived (yes/no). The outcome measure was the number of times the participant accurately reported vibration (actual or sham).

<u>Pinprick (Big Toe):</u> A disposable neurological examination pin (delivers a calibrated force of 40g) was applied to the big toe of the most affected side (if not different, the dominant side used). The procedure used alternating (in a non-regular pattern) active versus sham pinprick. The pin was applied 5 times. The outcome measure was the number of times the participant accurately reported the pinprick (0 to 5).

To ensure consistency of the neurologic evaluation, training and calibration across assessors was performed by the same neurologist, Dr. Shoichi Shimamoto.

<u>Additional Results:</u>

Table S1: Baseline Characteristics by Study Arm (n=43) for the analysis adjusted by time on study, baseline CIPN grade, and baseline score (as needed)

	Placebo	Active	Total	
	n = 22	n = 24	n = 46	p
**Age (Mean/SD)**	61.1 (9.4)	58.1 (8.2)	59.6 (8.8)	0.254

**Sex**				0.564
Male	3 (13.6%)	2 (8.3%)	5 (10.9%)	
Female	19 (86.4%)	22 (91.7%)	41 (89.1%)	
**Race**				0.623
White	15 (68.2%)	16 (66.7%)	31 (67.4%)	
Black	7 (31.8%)	7 (29.2%)	14 (30.4%)	
Other	0 (0%)	1 (4.2%)	1 (2.2%)	
**Ethnicity**				0.166
Hispanic	0 (0%)	2 (8.3%)	2 (4.4%)	
Not Hispanic	22 (100%)	22 (91.7%)	44 (95.6%)	
**Cancer Type**				0.628
Breast	14 (63.6%)	15 (62.5%)	29 (63.0%)	
Colon	3 (13.6%)	5 (20.8%)	8 (17.4%)	
Ovarian	4 (18.2%)	3 (12.5%)	7 (15.2%)	
Rectal	1 (4.5%)	0 (0%)	1 (2.2%)	
Uterine	0 (0%)	1 (4.2%)	1 (2.2%)	
**Chemotherapy**				0.698
Carboplatin	2 (9.1%)	1 (4.2%)	3 (6.5%)	
Cisplatin	1 (4.5%)	0 (0%)	1 (2.2%)	
Docetaxel	0 (0%)	2 (8.3%)	2 (4.4%)	
Oxaliplatin	4 (18.2%)	4 (16.7%)	8 (17.4%)	
Paclitaxel	6 (27.3%)	8 (33.3%)	14 (30.4%)	
Docetaxel, Carboplatin	0 (0%)	1 (4.2%)	1 (2.2%)	
Paclitaxel, Carboplatin	7 (31.8%)	7 (29.2%)	14 (30.4%)	
Paclitaxel, Doxorubicin	1 (4.5%)	1 (4.2%)	2 (4.4%)	
Paclitaxel, Doxorubicin, Carboplatin	1 (4.5%)	0 (0%)	1 (2.2%)	
**Time from Chemotherapy (Months, Median/IQR)**	2.9 (1.5-8.6)	4.2 (2.8-12)	3.4 (2.8-12.0)	0.553
**CIPN Sensory Grade**				0.086
2	22 (100%)	21 (87.5%)	43 (93.5%)	
3	0 (0%)	3 (12.5%)	3 (6.5%)	

Table S2. Side effects reported by participants with CTCAE severity scores (active group)

**Side Effect**	Active	CTCAE 1	CTCAE 2	CTCAE 3
Fatigue	9	3	6	0
GI Distress/Indigestion	4	1	3	0

Nausea	3	1	1	1
Skin Irritation	0	0	0	0
“Doesn’t like how it feels”	1	0	1	0
Lightheadedness	3	1	2	0
Sleep disturbance	2	0	2	0
Increased Sweating	1	1	0	0
Positional vertigo	1	0	1	0
Increased appetite	2	0	2	0
Feels “foggy”	1	0	1	0
Hot flashes	2	0	2	0
Dry Eyes/Mouth	0	0	0	0
Migraine/Headaches	0	0	0	0

Table S3. Side effects reported by participants with CTCAE severity scores (placebo group)

**Side Effect**	Placebo	CTCAE 1	CTCAE 2	CTCAE 3
Fatigue	10	1	7	2
GI Distress/Indigestion	2	0	2	0
Nausea	1	0	1	0
Skin Irritation	1	0	1	0
“Doesn’t like how it feels”	1	0	1	0
Lightheadedness	1	0	1	0
Sleep disturbance	3	1	2	0
Increased Sweating	0	0	0	0
Positional vertigo	1	0	1	0
Increased appetite	1	0	1	0
Feels “foggy”	0	0	0	0
Hot flashes	0	0	0	0
Dry Eyes/Mouth	1	0	1	0
Migraine/Headaches	1	0	1	0

Table S4. Sensitivity Analysis of Participants Who Finished Chemotherapy 18 months of Less at Baseline

	Placebo n = 17		CBD n = 21			
	Baseline	Change	Baseline	Change	p	Cohen's D
**Sensory**	44.2 (19.3)	8.9 (10.4)	41.1 (21.6)	13.9 (13.2)	0.233	-0.41
**Motor**	33.9 (26.8)	6.7 (18.3)	33.8 (24.5)	11.0 (10.4)	0.387	-0.3

Table S5. Sensitivity Analysis Adjusted Mixed Effects

	Adjusted Main Effects	
	Coeff. (95% CI)*	p
**Sensory**		
CBD	-13.4 (-25.4, -1.3)	0.034
**Motor**		
CBD	-10.1 (-24.3, 4.2)	0.166
**Tingling and Numbness**		
CBD	-14.7 (-26.8, -2.5)	0.018
**Pain in Hands/Feet**		
CBD	-12.0 (-28.2, 4.3)	0.148

References Cited

Norman, Geoffrey R., Jeff A. Sloan, and Kathleen W. Wyrwich. 2003. "Interpretation of Changes in Health-Related Quality of Life: The Remarkable Universality of Half a Standard Deviation." *Medical Care* 41(5): 582–92. doi:10.1097/01.MLR.0000062554.74615.4C.

Simon, Natalie B., Michael A. Danso, Thomas A. Alberico, Ethan Basch, and Antonia V. Bennett. 2017. "The Prevalence and Pattern of Chemotherapy-Induced Peripheral Neuropathy among Women with Breast Cancer Receiving Care in a Large Community Oncology Practice." *Quality of Life Research: An International Journal of Quality of Life Aspects of Treatment, Care and Rehabilitation* 26(10): 2763–72. doi:10.1007/s11136-017-1635-0.
